# Supplementary material for: Systematic decomposition of sequence determinants governing CRISPR/Cas9 specificity
Source: Nat Commun. 2022 Jan 25;13:474. doi: 10.1038/s41467-022-28028-x (PMC8789861; doi:10.1038/s41467-022-28028-x)
Supplement: Supplementary file 1 — Supplementary Information [file 41467_2022_28028_MOESM1_ESM.pdf]

Supplementary Information

Systematic decomposition of sequence determinants governing CRISPR/Cas9 specificity

Rongjie Fu [#], Wei He [#], Jinzhuang Dou, Oscar D. Villarreal, Ella Bedford, Helen Wang, Connie Hou, Liang Zhang, Yalong Wang, Dacheng Ma, Yiwen Chen, Xue Gao, Martin Depken, Han Xu *

#These authors contributed equally to this work.

*Correspondence: Han Xu (hxu4@mdanderson.org)

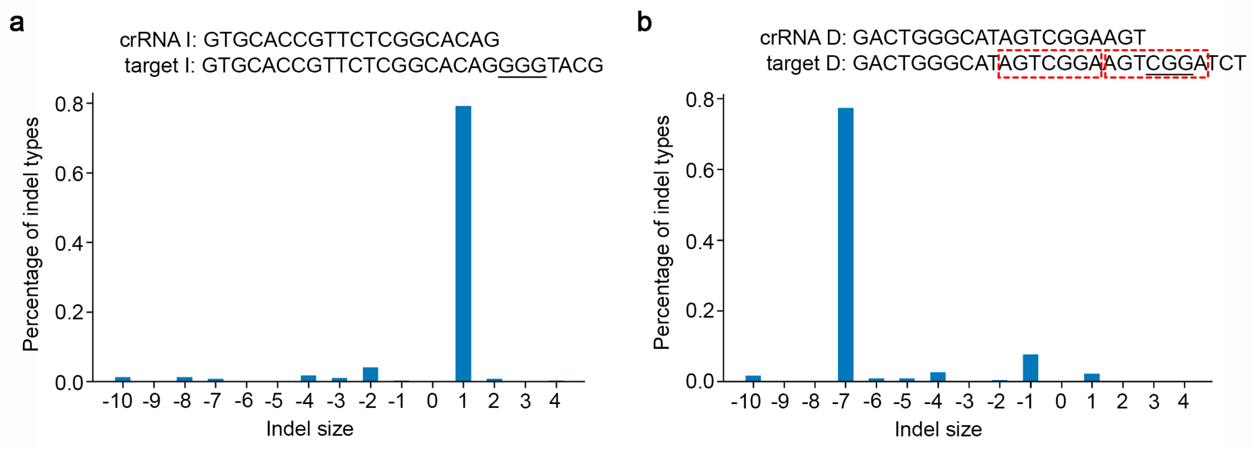

Supplementary Figure 1: **Indel distributions of two gRNAs for evaluation of dual-target system with distinct repair mechanisms upon double-strand breaks. (a)** gRNA I associated with dominating non-homologous end joining (NHEJ). **(b)** gRNA D associated with dominating microhomology-mediated end joining (MMEJ), where the microhomology sequences are highlighted in red rectangles. gRNA I and gRNA D were selected from a single-target system to model the outcomes of Cas9 mediated double-strand break repair, and the indel distributions shown here were calculated on the sequencing reads measured by this reported single-target system[1]. Source data for Supplementary Figure 1 are provided in the Source Data file.

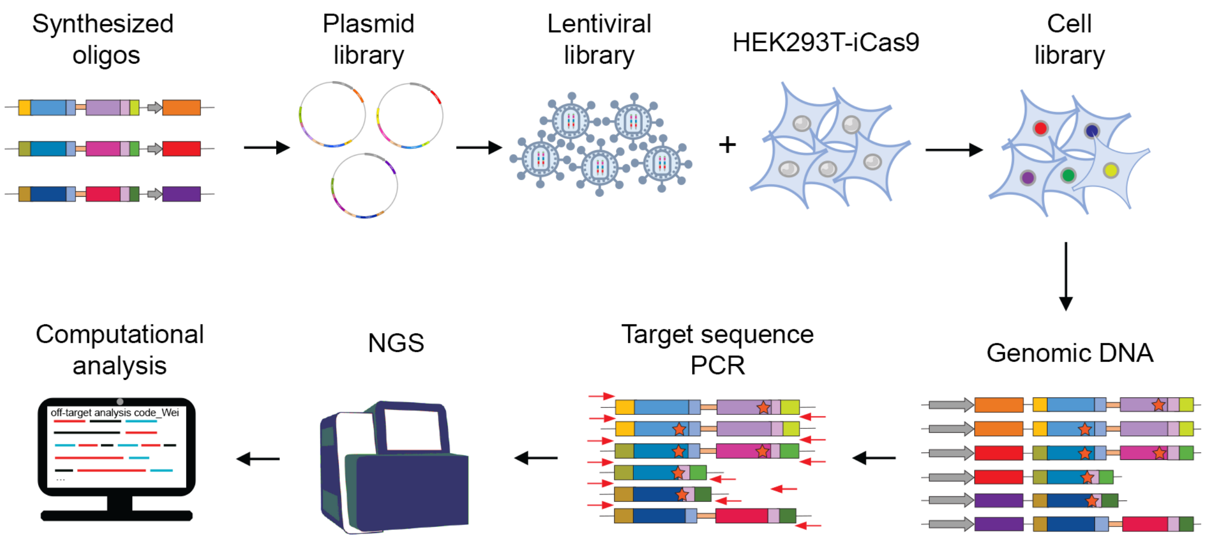

Supplementary Figure 2: **Schematic of experimental procedures to perform high-throughput screens with dual-target system.** The detailed sequence elements of each synthesized oligo library are shown in Supplementary Data 6 and Supplementary Note.

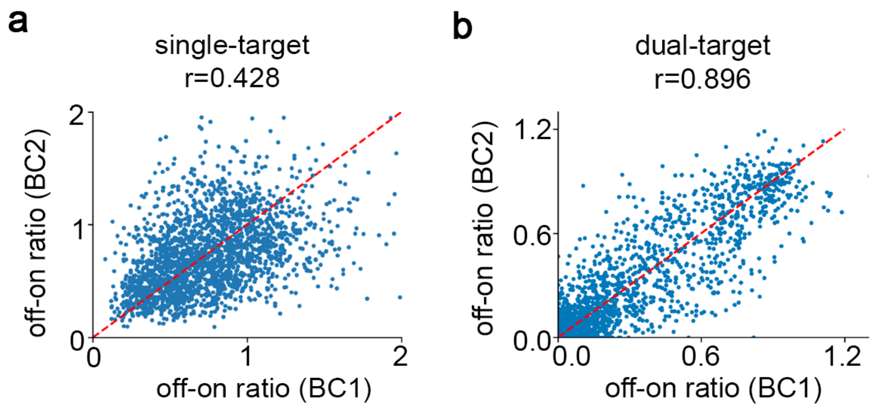

Supplementary Figure 3: **Correlation of off-on ratios between barcode sets.** Scatter plots showing the reproducibility of **(a)** single-target design and **(b)** dual-target design in the measurement of off-on ratios between non-overlapping barcode sets. Source data for Supplementary Figure 3 are provided in the Source Data file.

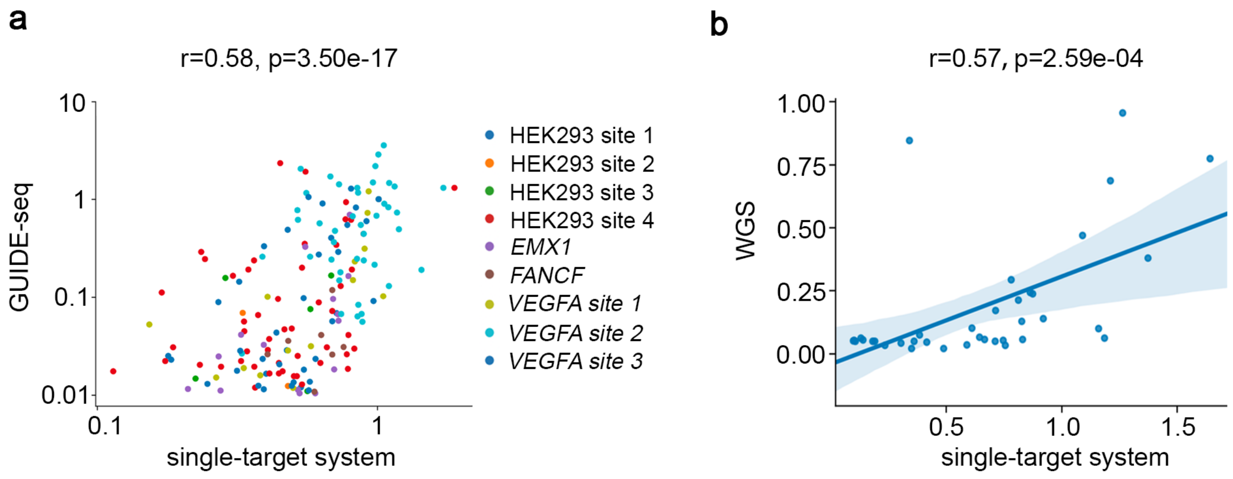

Supplementary Figure 4: Assessment of genomic off-targets by single-target system. Scatter plots showing the correlations between the off-on ratios estimated from the single-target system and **(a)** GUIDE-seq or **(b)** WGS, at the reported genomic off-target sequences. The p-values were calculated using Pearson correlation test. The shadow represents the 95% confidence interval. Source data for Supplementary Figure 4 are provided in the Source Data file.

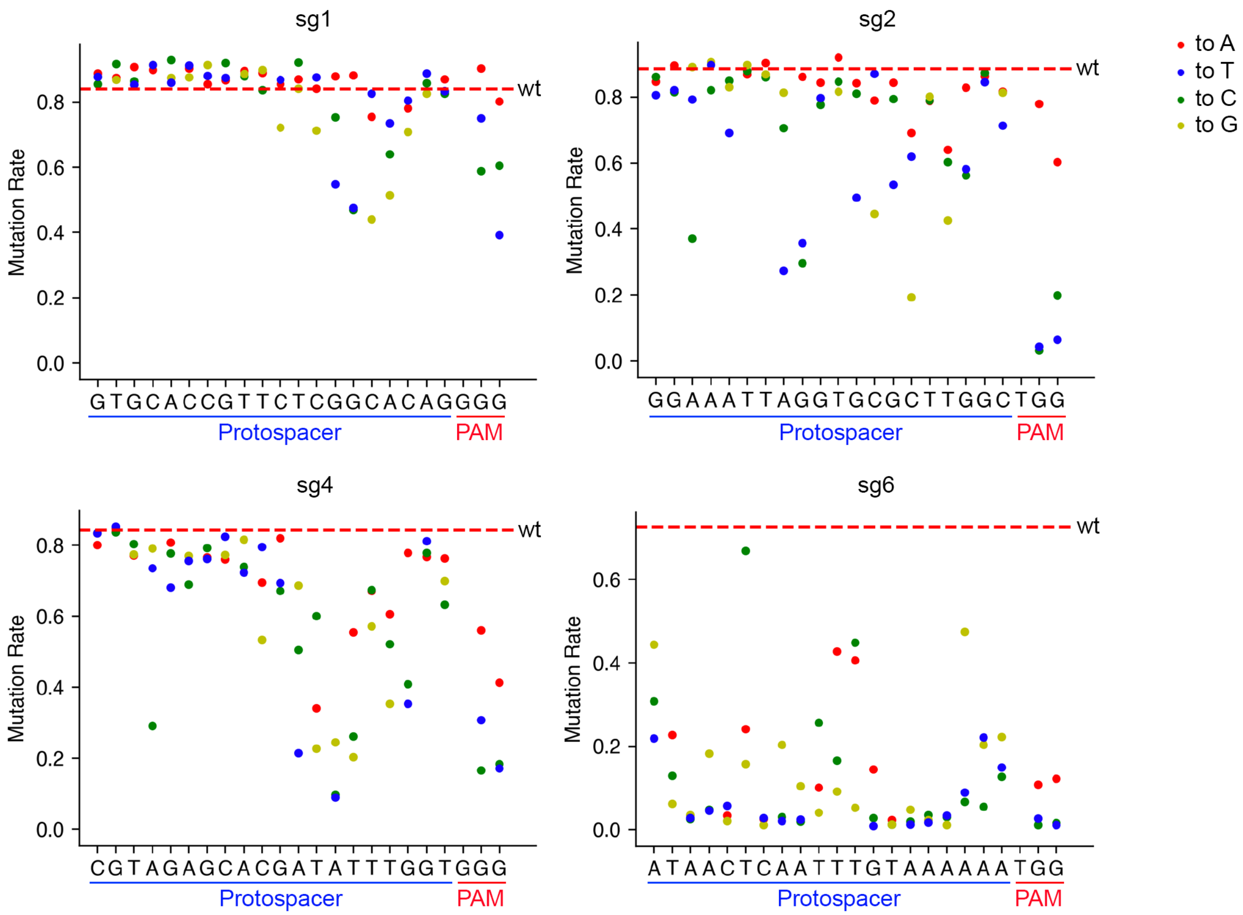

Supplementary Figure 5: **Single-mismatch profiles of 4 gRNAs.** The mutation rates at 1-MM target sequences of four example gRNAs that are associated with high GMT (sg1, sg2 and sg4) and low GMT (sg6). Each dot corresponds to a specific mismatched target. The red dashed line represents the mutation rate at the on-target sequence.

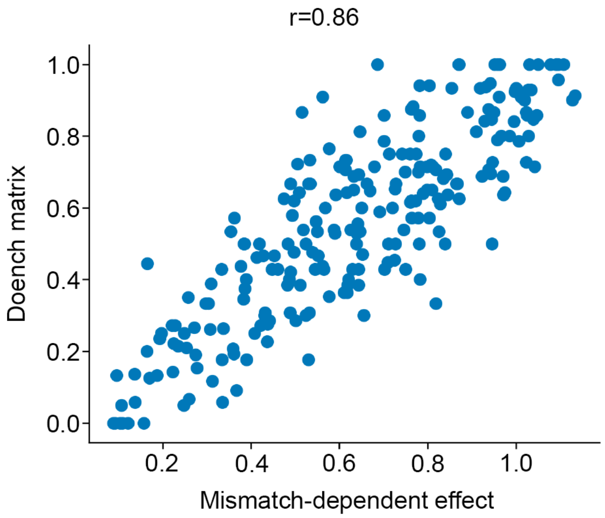

Supplementary Figure 6: **Correlation between 1-MM matrix and Doench's matrix**. A scatter plot showing the correlation of the mismatch-dependent effect between our and Doench's data[2]. Each dot represents the off-target effect for a certain type of single-mismatch at a specific position. Source data for Supplementary Figure 6 are provided in the Source Data file.

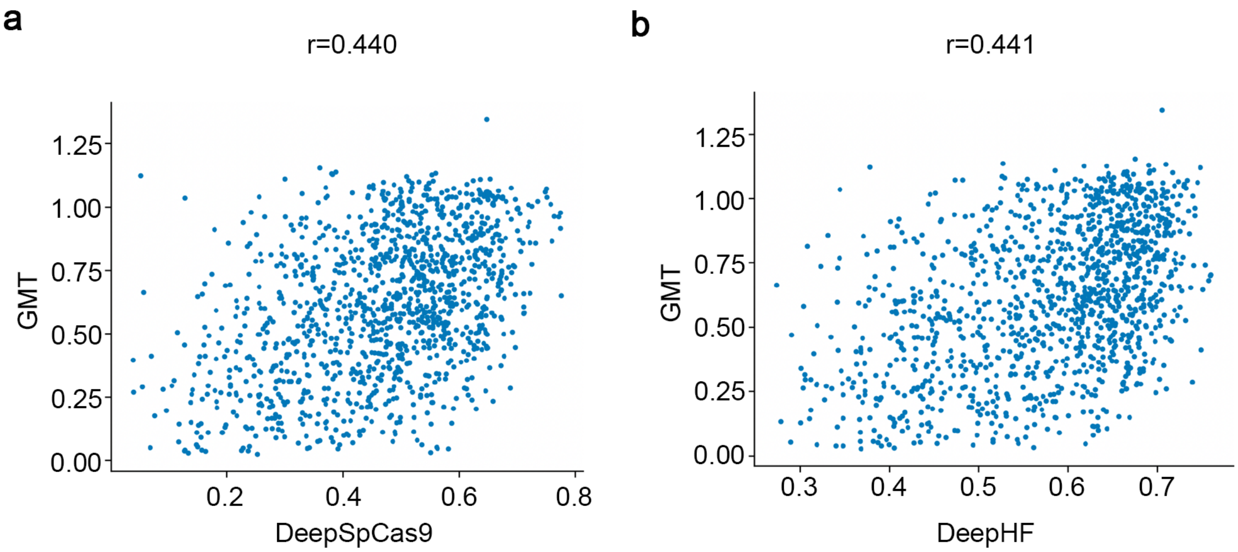

Supplementary Figure 7: **Correlation between GMT and gRNA activity**. Scatter plots showing the correlation between gRNA activity predicted by **(a)** DeepSpCas9[3] and **(b)** DeepHF[4] and guide-intrinsic mismatch tolerance (GMT). Each dot represents a gRNA designed in the dual-target library. Source data for Supplementary Figure 7 are provided in the Source Data file.

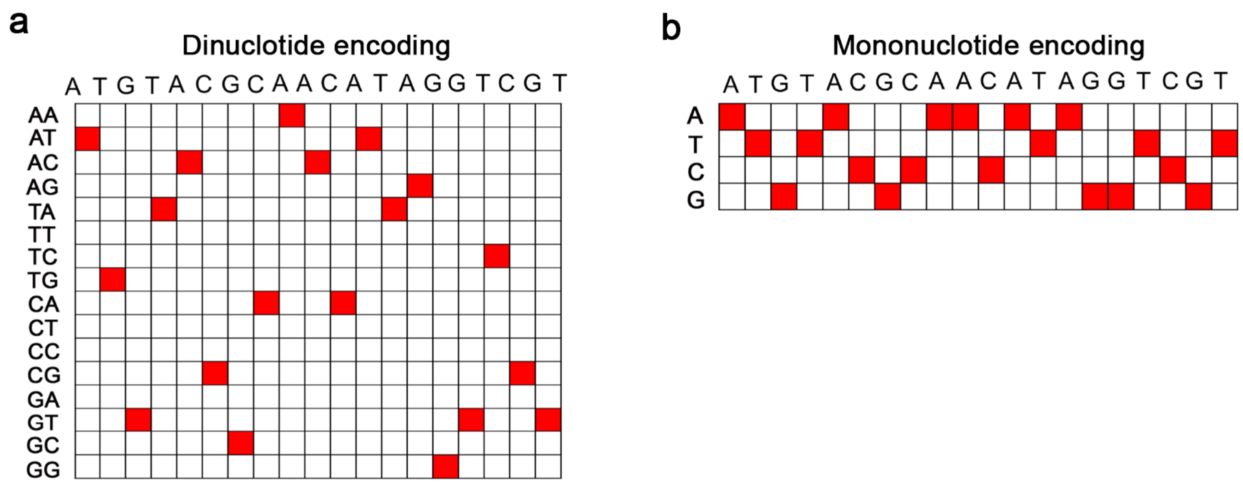

Supplementary Figure 8: **Schematic of two different data encoding approaches to vectorize gRNA sequence as inputs for CNN model. (a)** Dinucleotide encoding approach. **(b)** Mononucleotide encoding approach.

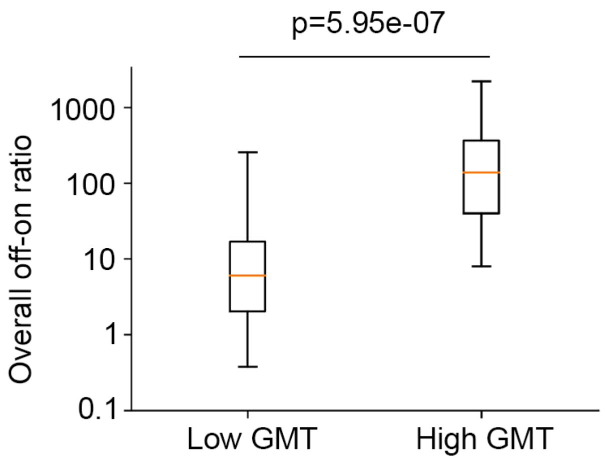

Supplementary Figure 9: **Comparison of the specificity between gRNAs classified into high and low GMT**. A boxplot showing the comparison of the overall off-on ratio between gRNAs with high GMT (top 25%, n=27) and low GMT (bottom 25%, n=27) in the CHANGE-seq dataset[5], the p-value was computed using the two-tailed Mann-Whitney U-test. The box plot displays a median line, interquartile range boxes and min to max whiskers. Source data for Supplementary Figure 9 are provided in the Source Data file.

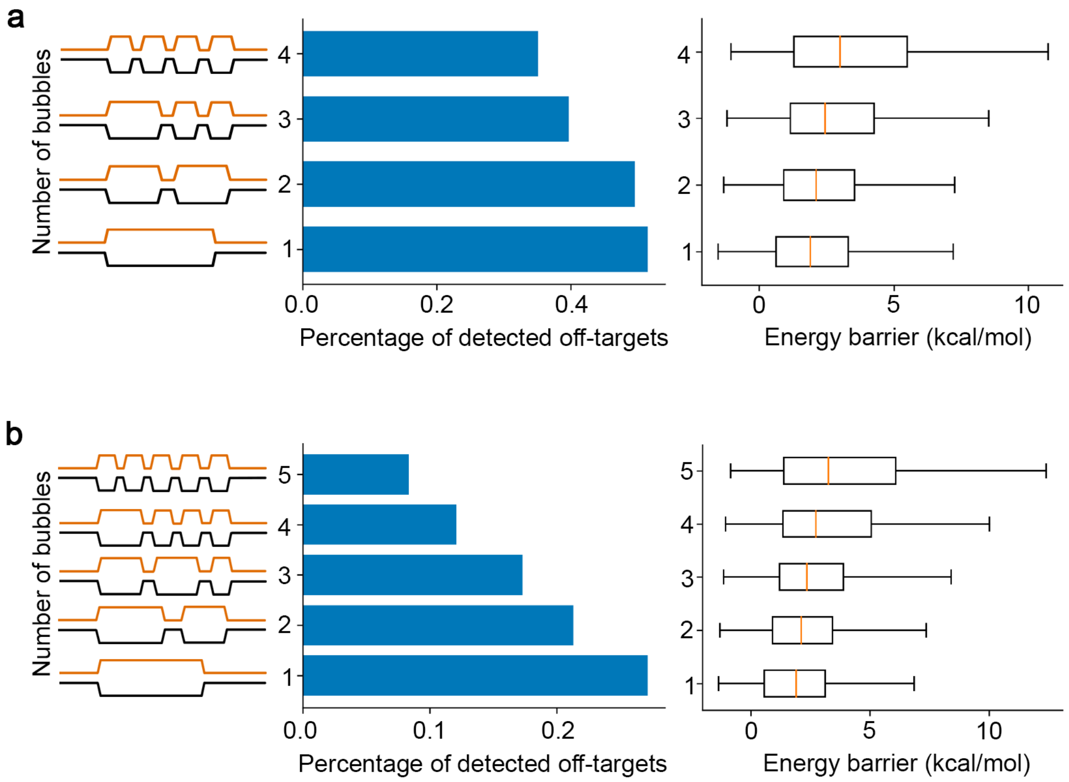

Supplementary Figure 10: **Correlation between bubble numbers and off-target effects.** Bar charts showing the percentage of off-target sites with different numbers of bubbles detected by CHANGE-seq [5]. Box plots showing the distribution of energy barrier computed from cumulative dinucleotide base-stacking energy changes between 1,000,000 random gRNA sequences and corresponding target sequences with randomly introduced bubbles. **(a)** Analyses on off-target sites harboring 4 mismatches. **(b)** Analyses on off-target sites harboring 5 mismatches. The box plots display a median line, interquartile range boxes and min to max whiskers. Source data for Supplementary Figure 10 are provided in the Source Data file.

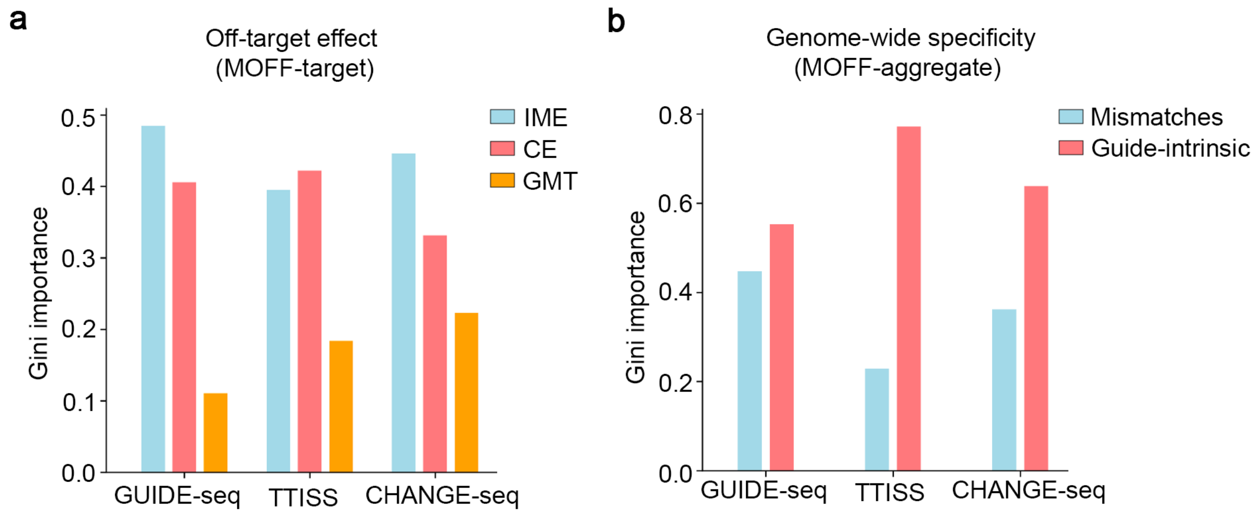

Supplementary Figure 11: **Comparison of feature importance in MOFF-target and MOFF-aggregate. (a)** Gini importance of IME, CE and GMT in MOFF-target. **(b)** Gini importance of mismatch-dependent effect and GMT in MOFF-aggregate. Source data for Supplementary Figure 11 are provided in the Source Data file.

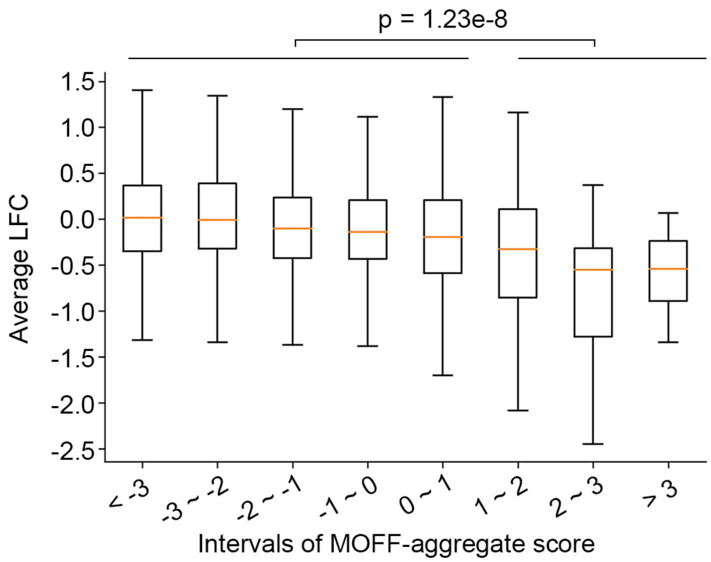

Supplementary Figure 12: **Correlation between MOFF-aggregate score and gRNA dropout effects in high-throughput CRISPR/Cas9 screen**. A box plot comparing the average log-fold change (LFC) of gRNAs targeting non-essential genes in GeCKO-v2 dataset across different MOFF-aggregate score intervals. The p-value was calculated using the two-tailed Mann-Whitney U-test comparing the LFC of gRNAs with MOFF-aggregate scores smaller and larger than 1. The data represent n = 236, 284, 496, 533, 382, 124, 20, and 4 gRNAs in each score interval from left to right. The box plot displays a median line, interquartile range boxes and min to max whiskers. Source data for Supplementary Figure 12 are provided in the Source Data file.

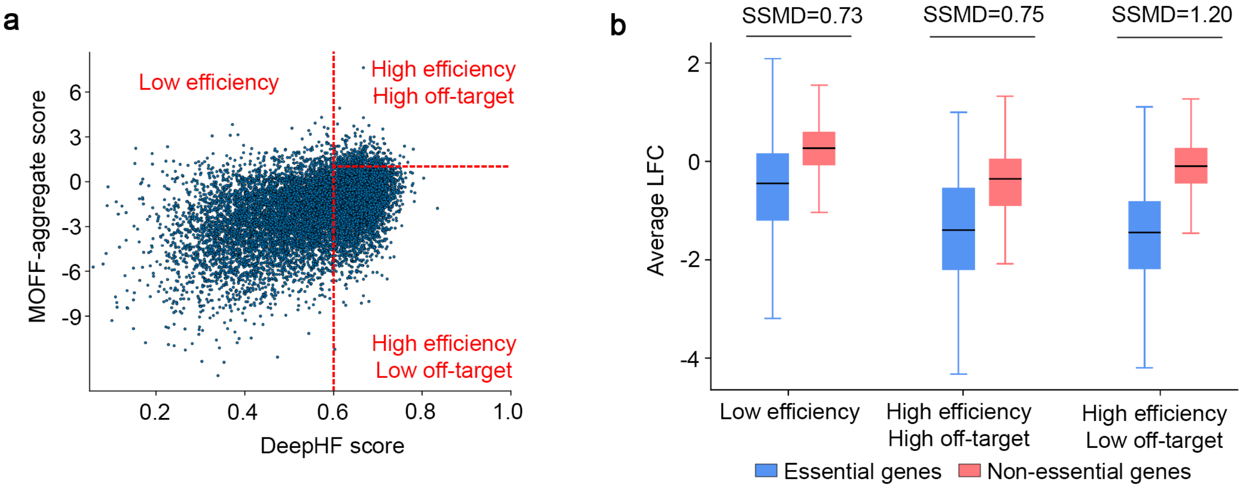

Supplementary Figure 13: **Application of MOFF for gRNA selection. (a)** A scatter plot showing the categorization of 11,701 gRNAs targeting 1,246 core essential genes and 758 non-essential genes in the GeCKO-v2 library, based on gRNA activity (DeepHF score, x-axis) and gRNA specificity (MOFF-aggregate score, y-axis). gRNAs were classified into 3 different categories: Low efficiency (DeepHF score < 0.6), High efficiency High off-target (DeepHF score > 0.6 and MOFF-aggregate > 1) and High efficiency Low off-target (DeepHF score > 0.6 and MOFF-aggregate < 1). **(b)** A box plot comparing the average log-fold change (LFC) of gRNAs targeting essential and non-essential genes among different gRNA categories. The differences between two groups within each category were measured by strictly standardized mean difference (SSMD). The data represents n = 4,176 essential, and 2,248 non-essential gRNAs in “low efficiency” group, n = 226 essential, and 148 non-essential gRNAs in “high efficiency high off-target” group, and n = 2,968 essential, and 1,936 non-essential gRNAs in “high efficiency low off-target” group. SSMD scores are computed as the measure of effect size in the screens. The box plot displays a median line, interquartile range boxes and min to max whiskers. Source data for Supplementary Figure 13 are provided in the Source Data file.

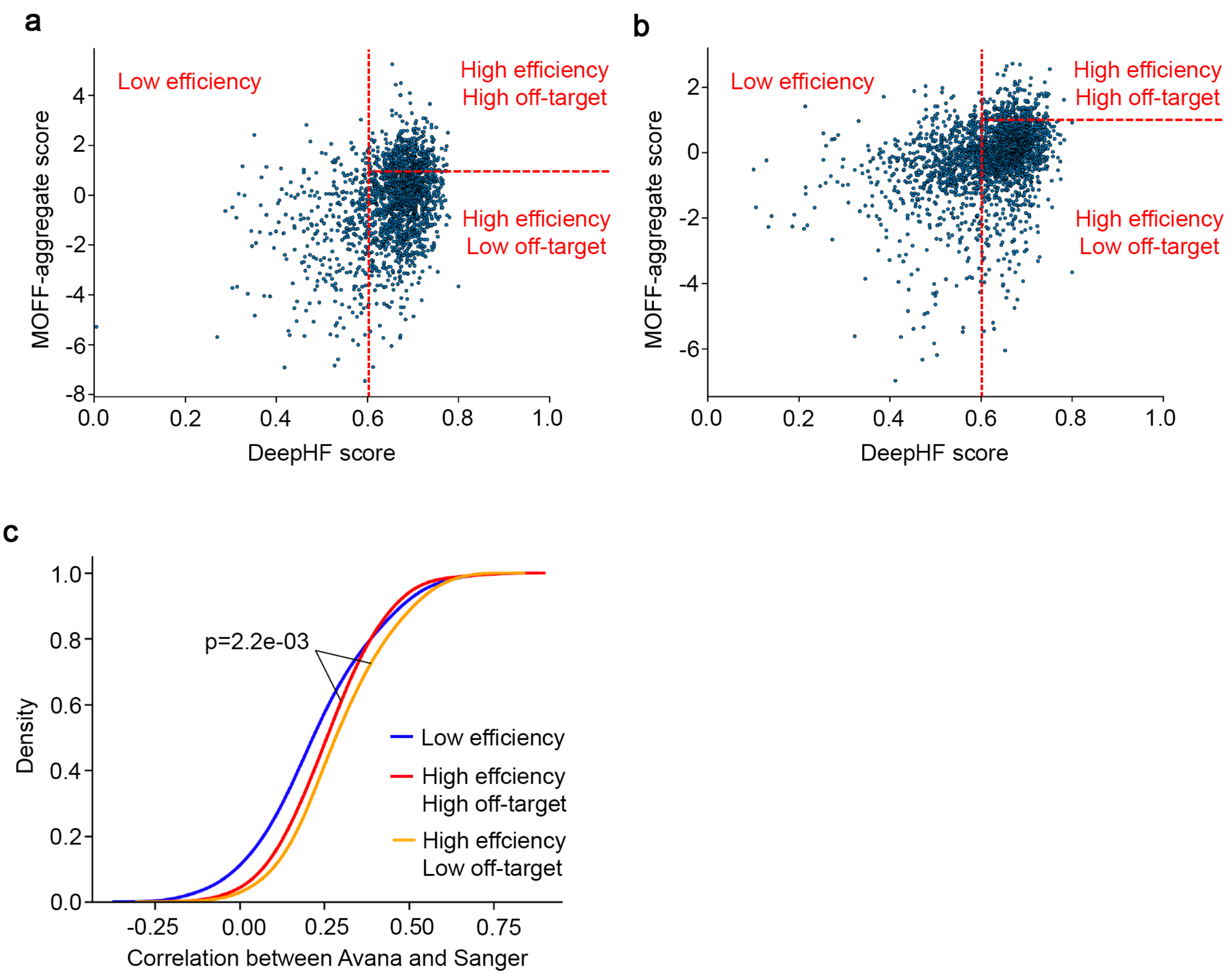

Supplementary Figure 14: **Comparison of the consistency between Avana and Sanger dataset among different gRNA categories. (a, b)** Scatter plots showing the categorization of gRNAs targeting 529 cell-specific essential genes in the **(a)** Avana and **(b)** Sanger libraries, based on gRNA activity (DeepHF score, x-axis) and gRNA specificity (MOFF-aggregate score, y-axis). The red dashed lines used for gRNA classifications represent MOFF aggregates score = 1 (horizontal) and DeepHF score = 0.6 (vertical), respectively. **(c)** Cumulative distribution of correlations between gRNAs targeting cell-specific essential genes in the Avana and Sanger datasets within different categories. Pearson correlation was adopted to measure the correlation of log-fold change (LFC) caused by gRNAs from two datasets targeting the same genes across different cell lines. The p-value was calculated using Two-sample Kolmogorov-Smirnov test (two-tailed). Source data for Supplementary Figure 14 are provided in the Source Data file.

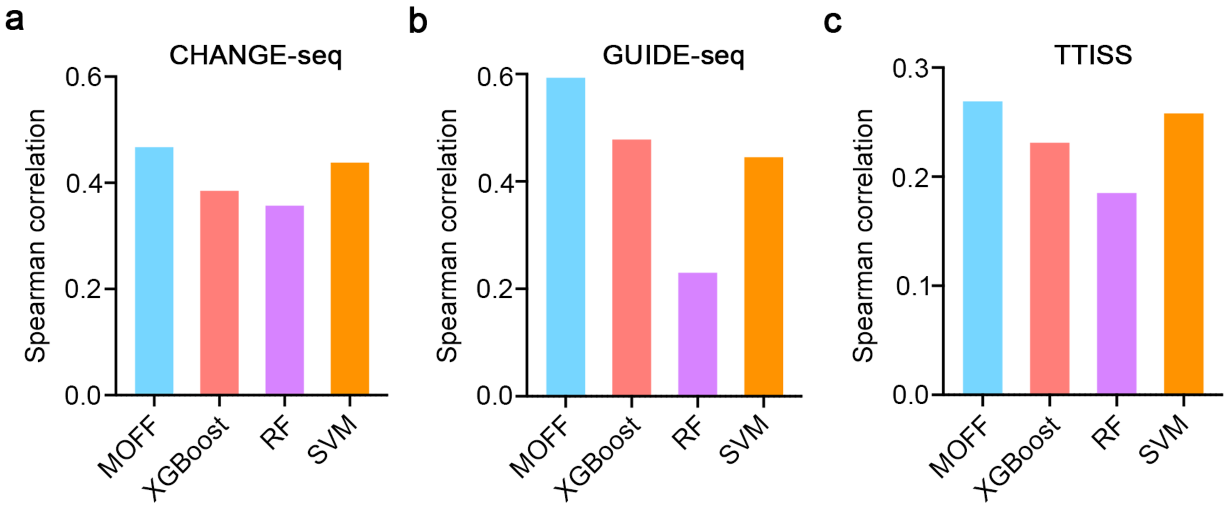

Supplementary Figure 15: Comparison of MOFF to traditional machine learning models. Bar charts showing the correlations between predicted off-target effects and measured off-on ratios in three test datasets. The MOFF prediction is compared to three machine learning models, Gradient Boosted Tree (XGBoost), Random Forest Regressor (RF), and Support Vector Machine (SVM). A consistent encoding method storing on- to off-target sequence nucleotides at each position was used as input. All the models were trained on the same dataset as MOFF (the dual-target dataset in this paper) and validated on the public datasets from **(a)** CHANGE-Seq, **(b)** GUIDE-Seq, and **(c)** TTISS. Because the training dataset includes <=3 mismatches in the design, the test datasets were filtered to include the off-target sites with <=3 mismatches only. Source data for Supplementary Figure 15 are provided in the Source Data file.

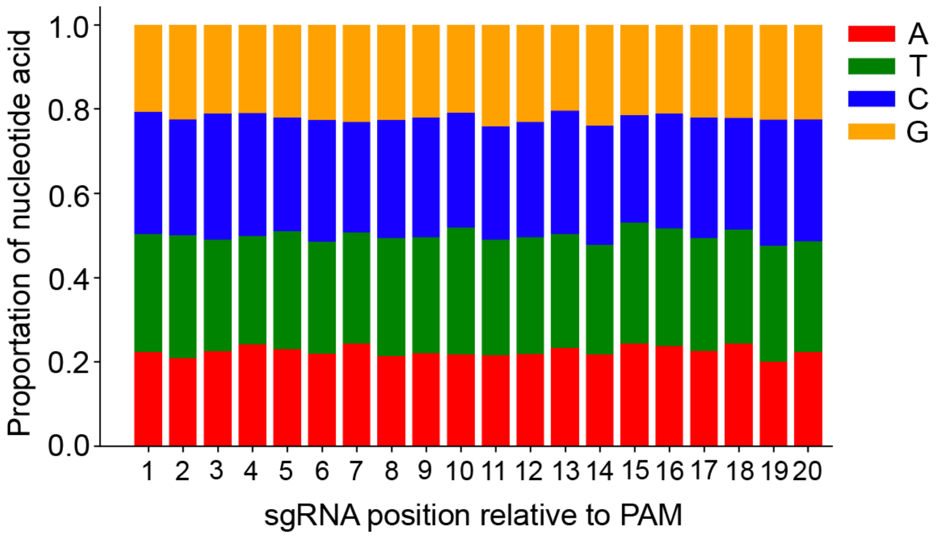

Supplementary Figure 16: Proportion of four nucleotides at each position for randomly designed gRNAs in the dual-target dataset. A bar chart showing the roughly evenly distributed frequency of four nucleotides (A, G, C and T) at each position for randomly designed gRNAs. Source data for Supplementary Figures 16 are provided in the Source Data file.

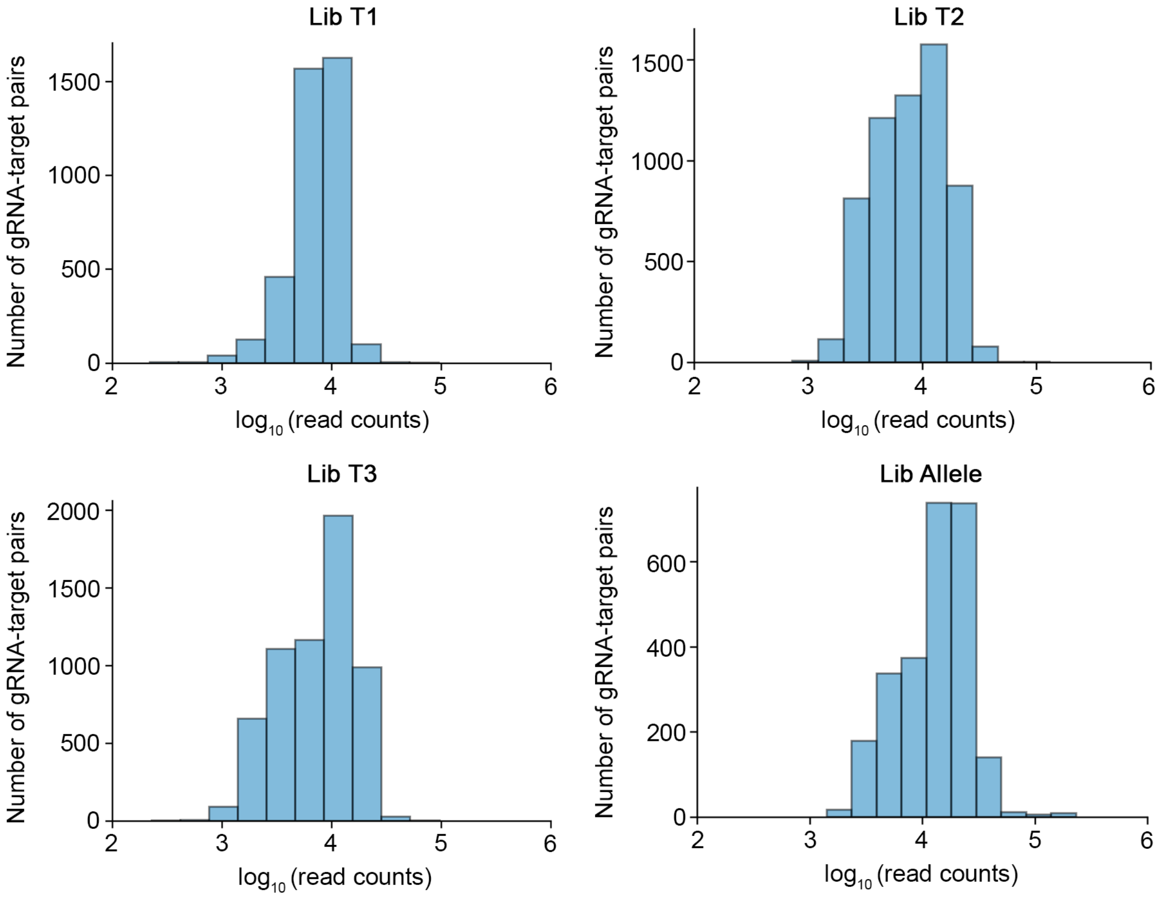

Supplementary Figure 17: Sequencing depths of the experiments with dual-target design. Histograms showing distributions of the number of gRNA-target pairs (y-axis) with a given sequencing depth (x-axis) across the experiments with dual-target design.

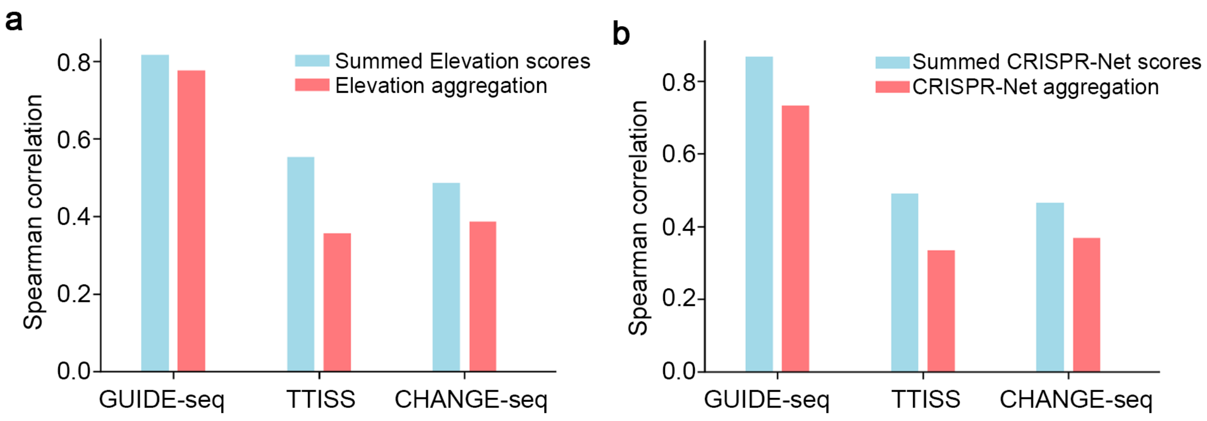

Supplementary Figure 18: Comparison of the performance of genome-wide specificity prediction using unweighted summation (blue bars) and weighted summation (red bars). The comparison was based on two published off-target prediction software, **(a)** Elevation[6] and **(b)** CRISPR-Net[7]. The test datasets were generated by GUIDE-seq, TTISS, and CHANGE-seq, respectively. **"**Summed Elevation scores" and "Summed CRISPR-Net scores" (blue bars) refer to unweighted summation of off-target effects at all possible off-target sites (mismatch <=6) in the genome. "Elevation aggregation" and "CRISPR-Net aggregation" (red bars) are based on weighted summation as implemented in the two software. The weights in the weighted approach were trained based on viability screening data, thus the performance of weighted sum is degraded when tested on the three datasets that were generated by off-target detection methods. Source data for Supplementary Figure 18 are provided in the Source Data file.

Supplementary Note

Sequence components of each oligonucleotide in different designed libraries:

(a) Paired gRNA and dual-target libraries (T1, T2, T3 and Allele)

AAGATAGTGCAGGAACACnnnnnnnnnnNNNNNNNNNNNNNNNNNNNNNGGnnnnnnnnnnnnn
nnNNNNNNNNNNNNNNNNNNNNNGGnnnnnnnnnnnnnnnAGCTTGGCGTAACTAGATCT**AAT**
ATTGTGGAAAGGACGAAACACCgNNNNNNNNNNNNNNNNNNNNGTTTTAGAGCTAGAAAT
AGC

(b) Paired gRNA and single-target library (S1)

AAGATAGTGCAGGAACACnnnnnnnnnnNNNNNNNNNNNNNNNNNNNNNGGnnnnnnnnnnnnn
nnnnnnnAGCTTGGCGTAACTAGATCT**AATATT**GTGGAAAGGACGAAACACCgNNNNNNNNN
NNNNNNNNNNNGTTTTAGAGCTAGAAATAGC

(c) Target-only libraries (sg1, sg2, sg3, sg4, sg5 and sg6)

CTAGATCTTGAGACAAATGGTTAATnnnnnnnnnnNNNNNNNNNNNNNNNNNNNNNGGNNNNn
nnnnnnnnnnnnnnnnnnnATTAACAGTATTCATCCACAATTTTAA

Annotations for each sequence components:

1) Primer binding sequence

2) Cloning linker sequence (containing a vector homology sequence, an SspI enzyme recognition site and an hU6 homology sequence)

3) **SspI enzyme recognition site**

4) Barcode 1

5) Barcode 2

6) Off-target sequence (Library T1, T2, T3 and Allele) / Target sequence (Library S1, sg1, sg2, sg3, sg4, sg5 and sg6)

7) On-target sequence (Library T1, T2, T3 and Allele)

8) gRNA sequence

9) Linker sequence to segregate two targets

Supplementary References

1. Allen, F. et al. Predicting the mutations generated by repair of Cas9-induced double-strand breaks. *Nat Biotechnol* (2018).
2. Doench, J.G. et al. Optimized sgRNA design to maximize activity and minimize off-target effects of CRISPR-Cas9. *Nat Biotechnol* **34**, 184-191 (2016).
3. Kim, H.K. et al. SpCas9 activity prediction by DeepSpCas9, a deep learning-based model with high generalization performance. *Sci Adv* **5**, eaax9249 (2019).
4. Wang, D. et al. Optimized CRISPR guide RNA design for two high-fidelity Cas9 variants by deep learning. *Nat Commun* **10**, 4284 (2019).
5. Lazzarotto, C.R. et al. CHANGE-seq reveals genetic and epigenetic effects on CRISPR-Cas9 genome-wide activity. *Nat Biotechnol* **38**, 1317-1327 (2020).
6. Listgarten, J. et al. Prediction of off-target activities for the end-to-end design of CRISPR guide RNAs. *Nat Biomed Eng* **2**, 38-47 (2018).
7. Lin, J.C., Zhang, Z.L., Zhang, S.X., Chen, J.Y. & Wong, K.C. CRISPR-Net: A Recurrent Convolutional Network Quantifies CRISPR Off-Target Activities with Mismatches and Indels. *Adv Sci* **7** (2020).
