# Supplementary material for: Systematic decomposition of sequence determinants governing CRISPR/Cas9 specificity
Source: Nat Commun. 2022 Jan 25;13:474. doi: 10.1038/s41467-022-28028-x (PMC8789861; doi:10.1038/s41467-022-28028-x)
Supplement: Supplementary file 2 — Reporting Summary [file 41467_2022_28028_MOESM2_ESM.pdf]

nature portfolio

Corresponding author(s): Han Xu

Last updated by author(s): Dec 24, 2021

Reporting Summary

Nature Portfolio wishes to improve the reproducibility of the work that we publish. This form provides structure for consistency and transparency in reporting. For further information on Nature Portfolio policies, see our Editorial Policies and the Editorial Policy Checklist.

Statistics

For all statistical analyses, confirm that the following items are present in the figure legend, table legend, main text, or Methods section.

n/a	Confirmed	
☐	☒	The exact sample size (*n*) for each experimental group/condition, given as a discrete number and unit of measurement
☐	☒	A statement on whether measurements were taken from distinct samples or whether the same sample was measured repeatedly
☐	☒	The statistical test(s) used AND whether they are one- or two-sided<br>*Only common tests should be described solely by name; describe more complex techniques in the Methods section.*
☒	☐	A description of all covariates tested
☒	☐	A description of any assumptions or corrections, such as tests of normality and adjustment for multiple comparisons
☐	☒	A full description of the statistical parameters including central tendency (e.g. means) or other basic estimates (e.g. regression coefficient) AND variation (e.g. standard deviation) or associated estimates of uncertainty (e.g. confidence intervals)
☒	☐	For null hypothesis testing, the test statistic (e.g. *F*, *t*, *r*) with confidence intervals, effect sizes, degrees of freedom and *P* value noted<br>*Give P values as exact values whenever suitable.*
☒	☐	For Bayesian analysis, information on the choice of priors and Markov chain Monte Carlo settings
☒	☐	For hierarchical and complex designs, identification of the appropriate level for tests and full reporting of outcomes
☐	☒	Estimates of effect sizes (e.g. Cohen's *d*, Pearson's *r*), indicating how they were calculated

Our web collection on statistics for biologists contains articles on many of the points above.

Software and code

Policy information about availability of computer code

Data collection	Deep sequencing data were collected using Illumina MiSeq, Hiseq 3000 and Nextseq 500 sequencer from MDACC-Smithville Next Generation Sequencing Core. Sanger sequencing data for the allele-specific editing validations were obtained using ABI 3500 Genetic Analyzer from MDACC-Smithiville Molecular Biology Core. 18 cancer hotspot mutations were curated from Memorial Sloan Kettering Cancer Center (MSKCC) Cancer Hotspots (https://www.cancerhotspots.org) and cBioportal for Cancer Genomics (https://www.cbioportal.org). Avana, GeCKO-v2 and Sanger CRISPR screen data is collected from the Depmap portal (https://depmap.org/portal/download).
Data analysis	Synthego ICE v2 CRISPR Analysis tool (https://ice.synthego.com) was applied to analyze the indel frequencies from the Sanger sequencing data for the validations of allele-specific editing. MOFF was written in Python (version 3.7), implemented as an open source software downloadable from https://github.com/MDhewei/MOFF. DeepHF (http://www.deephf.com/#/cas9) was used to predict the gRNA efficiency and can be downloaded from https://github.com/izhangcd/DeepHF. CRISPRtiz was applied to predict the gRNA specificity, which is available at https://github.com/pinellolab/CRISPRitz. Five representative off-target prediction methods were compared to MOFF, which are available at: CFD (PMID: 26780180, Supplementary Table 19 provided in the original paper), Elevation (https://github.com/Microsoft/Elevation), CNN_std (https://github.com/MichaelLinn/off_target_prediction), CRISPR-Net (https://codeocean.com/capsule/9553651/tree/v1), and CRISPRoff (https://github.com/RTH-tools/crisproff).

For manuscripts utilizing custom algorithms or software that are central to the research but not yet described in published literature, software must be made available to editors and reviewers. We strongly encourage code deposition in a community repository (e.g. GitHub). See the Nature Portfolio guidelines for submitting code & software for further information.

Data

Policy information about availability of data

All manuscripts must include a data availability statement. This statement should provide the following information, where applicable:

- Accession codes, unique identifiers, or web links for publicly available datasets
- A description of any restrictions on data availability
- For clinical datasets or third party data, please ensure that the statement adheres to our policy

The raw sequencing data of screens generated from this study are available under NCBI Sequence Read Archive (SRA) (SRA code: PRJNA732904), which can be accessed at https://www.ncbi.nlm.nih.gov/bioproject/PRJNA732904. The raw and processed read counts of the screens generated from this study are provided as Supplementary Data files. Three datasets used in this study are available under NCBI SRA: GUIDE-seq (SRA code: SRP050338, https://www.ncbi.nlm.nih.gov/sra?term=SRP050338), TTISS (SRA code: PRJNA602092, https://www.ncbi.nlm.nih.gov/bioproject/PRJNA602092/), and CHANGE-seq (SRA code: PRJNA625995, https://www.ncbi.nlm.nih.gov/bioproject/?term=PRJNA625995). Data for Avana, GeCKO-v2 and Sanger CRISPR screen are obtained from the Depmap portal at https://depmap.org/portal/download. Source data are provided with this paper.

Field-specific reporting

Please select the one below that is the best fit for your research. If you are not sure, read the appropriate sections before making your selection.

☒ Life sciences ☐ Behavioural & social sciences ☐ Ecological, evolutionary & environmental sciences

For a reference copy of the document with all sections, see nature.com/documents/nr-reporting-summary-flat.pdf

Life sciences study design

All studies must disclose on these points even when the disclosure is negative.

Sample size	All the CRISPR/Cas9 screens (Lib T1, T2, T3, Allele, S1 and Lib sg1-sg6) have been conducted in 3 independent biological replicates. Detailed statistics for included gRNA numbers and classes can be found in Supplementary Data 14. All the gRNA infection experiments for allele-specific editing validation have been conducted in 2 independent biological replicates. All sample sizes were sufficient for the model training and for the following statistical tests.
Data exclusions	(1) Considering that the calculation of off-on ratios are associated with large variation with limited number of reads in the denominator, we filtered out gRNA-target pairs with less than 100 total edited reads at on-target site. The criterion was established after deep sequencing, depending on the read counts. The data from CRSIPR/Cas9 screens with Lib T1, T2, T3 and Allele were processed with this data exclusion criterion. (2) Considering that the calculation of indel rate are associated with large variation with limited number of edited reads, we filtered out gRNA-target pairs with less than 100 total edited reads. The criterion was established after deep sequencing, depending on the read counts. The data from CRSIPR/Cas9 screens with Lib S1 and sg1-sg6 were processed with this data exclusion criterion.
Replication	All the CRISPR/Cas9 screens (Lib T1, T2, T3, Allele, S1 and Lib sg1-sg6) have been conducted in 3 independent biological replicates and all the gRNA infection experiments for allele-specific editing validation have been conducted in 2 independent biological replicates. All attempts at replication were successful.
Randomization	We designed 7 off-target sequences for each randomly designed gRNA, including 3 random targets with 1 mismatch (1-MM), 3 with 2 mismatches (2-MM), and 1 with 3 mismatches (3-MM). The mismatches in the 2-MM and 3-MM sequences are the combinations of the mismatches in 1-MM sequences. For fair comparison of the performances between dual-target and single-target system, the same sets of gRNA-target pairs were included in library T1 and S1. For the allele-specific editing validations, 3 mismatched gRNAs were selected according to the allele-editing screen results and were compared to the perfect and truncated gRNAs.
Blinding	Cells used to conduct screens and allele-specific editing validation experiments were grown under identical conditions. Blinding was not used.

Reporting for specific materials, systems and methods

We require information from authors about some types of materials, experimental systems and methods used in many studies. Here, indicate whether each material, system or method listed is relevant to your study. If you are not sure if a list item applies to your research, read the appropriate section before selecting a response.

Materials & experimental systems

n/a	Involved in the study
☒	☐ Antibodies
☐	☒ Eukaryotic cell lines
☒	☐ Palaeontology and archaeology
☒	☐ Animals and other organisms
☒	☐ Human research participants
☒	☐ Clinical data
☒	☐ Dual use research of concern

Methods

n/a	Involved in the study
☒	☐ ChIP-seq
☒	☐ Flow cytometry
☒	☐ MRI-based neuroimaging

Eukaryotic cell lines

Policy information about cell lines

Cell line source(s)	HEK293T cells, from American Type Culture Collection (ATCC)
Authentication	Cells were not authenticated.
Mycoplasma contamination	Cells were routinely tested for being free of mycoplasma contamination using MycoAlert™ Mycoplasma Detection Kit (Lonza).
Commonly misidentified lines (See ICLAC register)	HEK293T cells were purchased from ATCC and are not listed in the ICLAC.
