# Supplementary material for: Systematic decomposition of sequence determinants governing CRISPR/Cas9 specificity
Source: Nat Commun. 2022 Jan 25;13:474. doi: 10.1038/s41467-022-28028-x (PMC8789861; doi:10.1038/s41467-022-28028-x)
Supplement: Supplementary file 11 — Description of Additional Supplementary Files [file 41467_2022_28028_MOESM11_ESM.pdf]

**Title:** Supplementary Data 1:
**Description:** Screening data of all gRNA-target pairs for three libraries (Lib T1-3) using dual-target system.

**Title:** Supplementary Data 2:
**Description:** Screening data of all gRNA-target pairs for one library (Lib S1) using singletarget system.

**Title:** Supplementary Data 3:
**Description:** Screening data of six libraries (Lib sg1-6) using single-target system.

**Title:** Supplementary Data 4:
**Description:** Screening data of all the 1-mismatch gRNAtarget pairs in three dual-target libraries, which were used for the decomposition of 1-mismatchdependent effects and guideintrinsic mismatch tolerance (GMT).

**Title:** Supplementary Data 5:
**Description:** Screening data for allele-specific editing library (Lib Allele) using dual-target system.

**Title:** Supplementary Data 6:
**Description:** Summary of the detailed components in each designed library.

**Title:** Supplementary Data 7:
**Description:** Oligos and primers used for experiments.
